# Serum Claudin-5 and Peripheral Inflammation in Major Depressive Disorder: A Case-Control Study with Focus on Suicidal Ideation

**DOI:** 10.31083/AP46843

**Published:** 2026-02-25

**Authors:** Nurbanu Keskin, Mahmut Selçuk, Ercan Saruhan

**Affiliations:** ^1^Department of Psychiatry, Muğla Sıtkı Koçman University Faculty of Medicine Kötekli, 48000 Menteşe/Muğla, Türkiye; ^2^Department of Medical Biochemistry, Muğla Sıtkı Koçman University Faculty of Medicine Kötekli, 48000 Menteşe/Muğla, Türkiye

**Keywords:** blood–brain barrier, Claudin-5, inflammation, major depressive disorder, suicidal ideation

## Abstract

**Background:**

Major depressive disorder (MDD) has been increasingly associated with neuroinflammatory and neurovascular dysfunction. Claudin-5, a key tight junction protein essential for blood–brain barrier integrity, has an unclear role as a peripheral biomarker in MDD. This study examined serum Claudin-5 alongside systemic inflammatory indices—including the neutrophil-to-lymphocyte ratio (NLR), platelet-to-lymphocyte ratio (PLR), monocyte-to-lymphocyte ratio (MLR), the inflammation score (INFLA), and suicidal ideation in antidepressant-naive or medication-free (for at least three months) adult MDD patients.

**Method:**

73 antidepressant-naive or drug-free MDD patients and 74 age- and sex-matched healthy controls were enrolled. Depression severity and suicidality were assessed using the Hamilton Depression Rating Scale (HAM-D) and Beck Scale for Suicide Ideation (BSSI). Serum Claudin-5, C-reactive protein (CRP), and complete blood counts were measured, and inflammatory indices (NLR, PLR, MLR, INFLA) were calculated. Between-group comparisons, correlation analyses, and receiver operating characteristic (ROC) analyses were performed.

**Results:**

MDD patients showed significantly reduced Claudin-5 and elevated NLR, PLR, CRP, and INFLA scores compared with controls (all *p* < 0.05). Claudin-5 was not associated with symptom severity, suicidality, or inflammatory indices. ROC analysis for serum Claudin-5 indicated fair accuracy in distinguishing MDD from controls (AUC = 0.737) but limited value for predicting suicidal ideation (AUC = 0.628).

**Conclusion:**

Reduced serum Claudin-5 in untreated MDD may indicate relatively stable endothelial alterations rather than acute, state-dependent changes. Although Claudin-5 alone had limited prognostic value for suicidality, inflammatory indices—particularly NLR and INFLA—showed stronger associations. Integrating vascular and immune biomarkers may enhance biological stratification and suicide risk assessment in depression and guide future multimodal studies.

## Main Points

∙ Serum Claudin*-*5 levels were significantly reduced in drug-free or antidepressant-naive major depressive disorder (MDD) patients compared to healthy controls, indicating blood–brain barrier (BBB) disruption.

∙ Patients with MDD showed significantly higher neutrophil-to-lymphocyte ratio (NLR), platelet-to-lymphocyte ratio (PLR), C-reactive protein (CRP), and the inflammation score (INFLA), supporting a model of low-grade systemic inflammation in depression.

∙ Claudin-5 levels were not related to depression severity, suicidality, or inflammation, indicating relatively stable endothelial changes in MDD.

∙ NLR and INFLA score emerged as independent predictors of suicidal ideation in multivariate regression analysis.

∙ Combined use of Claudin-5 and inflammatory indices may provide complementary biological information for stratification and suicide risk assessment, rather than improving predictive performance.

## 1. Introduction

Major depressive disorder (MDD) is a complex psychiatric condition influenced by biological and environmental factors [1]. Among biological mechanisms, inflammation has gained particular attention for linking peripheral immune activation with central neurobiological alterations—including disrupted neuroimmune signaling, neurotransmission, and neurovascular integrity—that contribute to depressive symptoms [2, 3]. Dysregulated immune responses and elevated peripheral inflammation have been widely reported in depression and have been linked to poorer prognosis and reduced antidepressant response [1, 2]. Dysfunction of the blood–brain barrier (BBB) and neurovascular unit may mediate the effects of systemic inflammation on the central nervous system (CNS) [3, 4].

The BBB is a selective interface that maintains neural homeostasis by separating the CNS from systemic circulation. It consists of endothelial cells, pericytes, astrocytes, neurons, and microglia [5]. Tight junctions between endothelial cells regulate paracellular permeability. Claudin-5, the main transmembrane protein in endothelial tight junctions, is expressed throughout the vascular system but shows particularly high expression in brain microvascular endothelial cells, where it maintains BBB integrity [6, 7].

Disruption of Claudin-5 compromises BBB integrity, allowing peripheral cytokines such as interleukin-6 (IL-6), tumor necrosis factor-alpha (TNF-α), and interleukin-1 beta to enter the brain and trigger neuroinflammation and neuronal injury [8, 9].

The mechanisms underlying altered peripheral Claudin-5 levels remain debated. One hypothesis proposes a spillover mechanism in which BBB leakage releases tight-junction proteins into circulation [10, 11], while another suggests transcriptional downregulation, where inflammatory or epigenetic factors suppress Claudin-5 expression in CNS endothelial cells [12, 13]. Claudin-5 expression is modulated by pro-inflammatory cytokines, vascular endothelial growth factor, and glutamate (downregulation) and by estrogen, glucocorticoids, and glial cell line-derived neurotrophic factor (upregulation) [14, 15]. Because cerebrospinal fluid sampling is invasive, serum Claudin-5 provides a more accessible but indirect marker of BBB integrity. However, peripheral levels may only partially mirror central tight-junction status. Experimental and clinical evidence support this interpretation, showing that Claudin-5 dysregulation alters BBB permeability and contributes to neuroinflammatory changes in depression and related disorders [10, 11, 12, 13, 16, 17]. Such studies collectively support the use of serum Claudin-5 as an accessible, albeit indirect, indicator of BBB integrity.

Due to its tight-junction specificity, Claudin-5 has emerged as a key biomarker of neurovascular dysfunction in depression. Unlike general inflammatory markers, it directly reflects endothelial integrity. Preclinical studies show that Claudin-5 dysregulation increases BBB permeability and induces stress-related depressive phenotypes [13, 16]. Postmortem research has revealed reduced *Claudin-5* mRNA expression in the nucleus accumbens of unmedicated individuals with MDD, as demonstrated by Menard *et al*. [13], and summarized in subsequent reviews [16]. Nonetheless, there are few clinical studies assessing peripheral Claudin-5 levels in untreated adult MDD populations.

Animal models show that chronic social defeat stress downregulates Claudin-5 in brain regions such as the prefrontal cortex and nucleus accumbens, leading to increased BBB permeability and depressive-like behaviors [10, 12, 13]. Recent evidence also identifies Claudin-5 as a potential therapeutic target in depression and schizophrenia [5, 16, 17].

Moreover, BBB dysfunction and Claudin-5 downregulation have been linked to suicidal ideation, a major concern in MDD. Elevated IL-6 and TNF-α levels are observed in individuals with suicidal thoughts, suggesting that cytokine-induced BBB disruption may trigger glial activation and affective dysregulation [18, 19, 20].

Postmortem and experimental studies further confirm Claudin-5 reduction in the brains of suicide victims and stress-exposed animals showing depressive behaviors [12, 13].

Beyond molecular BBB markers, systemic inflammatory indices—such as the neutrophil-to-lymphocyte ratio (NLR), platelet-to-lymphocyte ratio (PLR), monocyte-to-lymphocyte ratio (MLR), and the inflammation score** (**INFLA)—have emerged as simple and inexpensive indicators of low-grade inflammation. Meta-analytic and clinical studies show that NLR, PLR, and MLR are elevated in depressive disorders and associated with symptom severity [21, 22]. These indices are also linked to suicidal ideation and behavior, suggesting systemic immune activation that compromises BBB integrity and interacts with Claudin-5 dysregulation in depression and suicidality [18, 19, 23].

Despite these findings, many previous studies included heterogeneous samples with ongoing treatment or medical comorbidities, limiting interpretability [23, 24, 25]. To date, no clinical study has simultaneously examined Claudin-5 with peripheral inflammatory markers—NLR, PLR, MLR, and the INFLA score—in relation to suicidal ideation in an untreated adult MDD population. This present study addresses that gap by evaluating Claudin-5 as a peripheral marker of neurovascular integrity alongside systemic inflammation and suicidality, compared with age- and sex-matched healthy controls.

By integrating molecular and immunological biomarkers in an untreated clinical cohort, this study aims to clarify how neurovascular and inflammatory mechanisms contribute to MDD. Such an approach may inform the development of multidimensional biomarkers and novel therapeutic targets beyond symptom-based frameworks. Here, it is hypothesized that serum Claudin-5 levels should be lower in MDD patients than in controls and correlate with both suicidal ideation and inflammatory indices (NLR, PLR, MLR, INFLA score).

## 2. Materials and Methods

### 2.1 Participants

A total of 147 participants were included: 73 patients diagnosed with MDD and 74 age- and sex-matched healthy controls. All patients were recruited from the Psychiatry Outpatient Clinic of Muğla Training and Research Hospital and were evaluated during an acute depressive episode. None were hospitalized at the time of assessment. All patients were medication-free for at least three months, and both illness duration and episode history were recorded during the Structured Clinical Interview for DSM-5 Disorders (SCID-5) clinical interview and review of medical history. Based on this information, patients were classified as either first-episode (antidepressant-naive) or recurrent (with previous episodes but currently drug-free). Controls were recruited from hospital staff and community volunteers, matched for age and sex, and were free from any current or past psychiatric disorders or systemic illnesses (confirmed by SCID-5).

### 2.2 Inclusion Criteria for the MDD Group

∙ Aged between 18 and 65 years.

∙ DSM-5 diagnosis of Major Depressive Disorder, confirmed by SCID-5.

∙ Drug-free status, defined as either antidepressant-naive or off all psychotropic and anti-inflammatory medications for at least three months.

∙ No acute or chronic infectious, autoimmune, cardiovascular, malignant, or metabolic diseases.

∙ No history of major physical trauma or surgery within the preceding three months.

∙ Literate and cognitively capable of completing self-report instruments (confirmed during the SCID-5 clinical interview through assessment of comprehension, attention, and communication).

∙ Not currently pregnant or breastfeeding.

∙ Provided written informed consent.

### 2.3 Inclusion Criteria for the Control Group

∙ Aged between 18 and 65 years.

∙ No current or lifetime psychiatric diagnosis (based on DSM-5 criteria, confirmed by SCID-5).

∙ No use of psychotropic or anti-inflammatory medications within the preceding three months.

∙ No significant medical illness, major trauma, or recent surgery.

∙ Not currently pregnant or breastfeeding.

∙ Literate and cognitively capable of completing self-report instruments (confirmed during the SCID-5 clinical interview through assessment of comprehension, attention, and communication).

∙ Provided written informed consent.

### 2.4 Rationale for the Three-Month Medication and Medical Washout Period

Participants were excluded if they had used any psychotropic or anti-inflammatory medications (e.g., antidepressants, antipsychotics, mood stabilizers, non-steroidal anti-inflammatory drugs (NSAIDs), or corticosteroids) or had experienced major physical trauma or surgery in the preceding three months. This restriction was implemented as both pharmacologic and physical factors significantly alter systemic inflammation and endothelial biomarkers.

Antidepressants and other psychotropic medications modulate cytokine production (e.g., IL-6, TNF-α), oxidative stress pathways, and neurovascular function, thereby normalizing or masking the inflammatory and endothelial abnormalities characteristic of untreated MDD. Similarly, NSAIDs and corticosteroids suppress cytokine levels such as IL-6 and C-reactive protein (CRP), which influence endothelial function for several weeks after use [26, 27]. These pharmacological effects could obscure intrinsic biomarker patterns linked to depression.

Additionally, tissue injury or surgical intervention activates immune responses and increases vascular permeability, with effects that may persist for months [28]. Therefore, a three-month washout period was applied to minimize residual influences of medications or recent systemic stressors and ensure that the measured biomarkers more accurately reflect the underlying pathophysiology of MDD.

### 2.5 Blinding

Clinical raters administering the Hamilton Depression Rating Scale (HAM-D) and Beck Scale for Suicide Ideation (BSSI) were independent psychiatrists blinded to the diagnostic status of participants, laboratory data, and study hypotheses. Group assignments (MDD vs. control) were coded by a research assistant not involved in assessment or analysis, and all rating sessions were conducted using standardized instructions without access to demographic or biochemical information. Laboratory personnel analyzing serum Claudin-5, CRP, and hematologic parameters were likewise blinded to all clinical information and received only anonymized sample codes. Data coding and blinding were maintained until completion of statistical analysis, after which group labels were re-linked by an independent investigator for final reporting.

### 2.6 Psychometric Instruments

#### 2.6.1 Structured Clinical Interview for DSM-5 Disorders (SCID-5)

All participants were evaluated using the SCID-5, a semi-structured diagnostic tool widely used in psychiatric research to ensure standardized assessment and diagnostic reliability. The Turkish validity and reliability study was conducted by Elbir *et al*. [29].

#### 2.6.2 Sociodemographic and Clinical Data Form (SCDF)

A semi-structured form developed by the researchers was used to record participants’ demographic and clinical characteristics, including age, gender, education, marital status, comorbidities, substance use, treatment history, and suicidal ideation or attempts.

#### 2.6.3 Hamilton Depression Rating Scale (HAM-D)

The HAM-D, developed by Hamilton (1960), is a clinician-rated scale assessing depression severity across 21 items, including mood, guilt, suicidality, sleep, somatic symptoms, and insight. Total scores (0–51) indicate severity: 0–7 = none, 8–15 = mild, 16–28 = moderate, ≥29 = severe. The Turkish version was validated by Akdemir *et al*. [30].

#### 2.6.4 Beck Scale for Suicide Ideation (BSSI)

Suicidal ideation was evaluated using both a clinical interview and the BSSI, a 21-item clinician-rated scale assessing passive and active suicidal thoughts, intent and planning. Items are scored 0–2 (total = 0–38), with higher scores indicating greater severity. Developed by Beck* et al*. [31], its Turkish version was validated by Ozcelik *et al*. [32].

The psychometric instruments (SCID-5, HAM-D, and BSSI) were administered face-to-face by board-certified psychiatrists experienced in the use of standardized rating scales and trained in the SCID-5. The same psychiatrists conducted all assessments in a consistent, blinded manner to ensure inter-rater reliability and procedural standardization across participants.

### 2.7 Biochemical Analysis

After a 12-hour overnight fast, venous blood samples were collected between 08:00 and 10:00 AM for complete blood count (CBC), CRP, and Claudin-5 analysis. CBC samples were drawn into ethylenediaminetetraacetic acid (EDTA) tubes and CRP/Claudin-5 samples into gel-separator tubes, then centrifuged at 1300 ×g (relative centrifugal force, RCF) for 15 minutes.

CBC was analyzed using an automated hematology analyzer (SP-50, Sysmex Corporation, Kobe, Japan). From CBC data, NLR, MLR, and PLR ratios were calculated. CRP levels were measured via immunoturbidimetry (Cobas c-701, Roche Diagnostics, Basel, Switzerland).

Serum Claudin-5 concentrations were determined by enzyme-linked immunosorbent assay using a commercial kit (BT LAB, Shanghai, China; Cat. No. E2303Hu; detection range 20–4500 ng/L, sensitivity 9.51 ng/L), following the manufacturer’s protocol.

The INFLA-score, originally proposed by Bonaccio *et al*. [33], combines CRP, white blood cell (WBC) count, platelet count, and granulocyte-to-lymphocyte ratio into a composite index of low-grade inflammation (range –16 to +16, higher values indicating greater inflammation). It has been validated in large population-based cohorts and shown to correlate with depressive symptoms, supporting its use as an integrated marker of systemic inflammation in depression [33, 34].

### 2.8 Statistical Analysis

Sample size estimation with G*Power 3.1 (Heinrich Heine University, Düsseldorf, Germany) indicated that 71 participants per group were required to detect a medium effect size (Cohen’s d = 0.5) with 80% power at α = 0.05. The final sample comprised 73 MDD patients and 74 controls (*n* = 147), meeting this criterion.

Analyses were conducted in IBM SPSS Statistics 26.0 (IBM Corp., Armonk, NY, USA). Descriptive data were summarized as mean ± SD, median (range), or percentages. Normality was assessed analytically. Between-group comparisons used Student’s *t*-test or Mann–Whitney *U* test, and categorical variables were analyzed with chi-square tests. One-way ANOVA or Kruskal–Wallis tests were applied for multi-group comparisons, and correlations were examined using Pearson or Spearman coefficients. Statistical significance was assumed for *p* < 0.05.

Multivariate logistic regression identified independent predictors of suicidal ideation within the MDD group. Analyses for depression severity (HAM-D) and diagnostic status (MDD vs. control) were not performed, as the former showed limited within-group variance and the latter was already examined in group comparisons. Receiver operating characteristic (ROC) analyses assessed the diagnostic accuracy of Claudin-5 and inflammatory indices for differentiating patients from controls and exploring their predictive value for suicidal ideation. Although group-level differences were nonsignificant, these exploratory analyses aimed to assess potential individual-level discriminative ability.

Variables included Claudin-5, NLR, INFLA score, and family history of psychiatric illness, selected *a priori* based on theoretical and empirical relevance and univariate associations (*p* < 0.10). Continuous variables were standardized before entry, and multicollinearity was checked using the Variance Inflation Factor (VIF), with values >5 indicating collinearity.

Effect sizes were interpreted according to Cohen’s criteria: small (d ≈ 0.2, η^2^ ≈ 0.01, V ≈ 0.10), medium (d ≈ 0.5, η^2^ ≈ 0.06, V ≈ 0.30), and large (d ≥0.8, η^2^ ≥0.14, V ≥0.50) [35].

## 3. Results

**Fig. 1.**
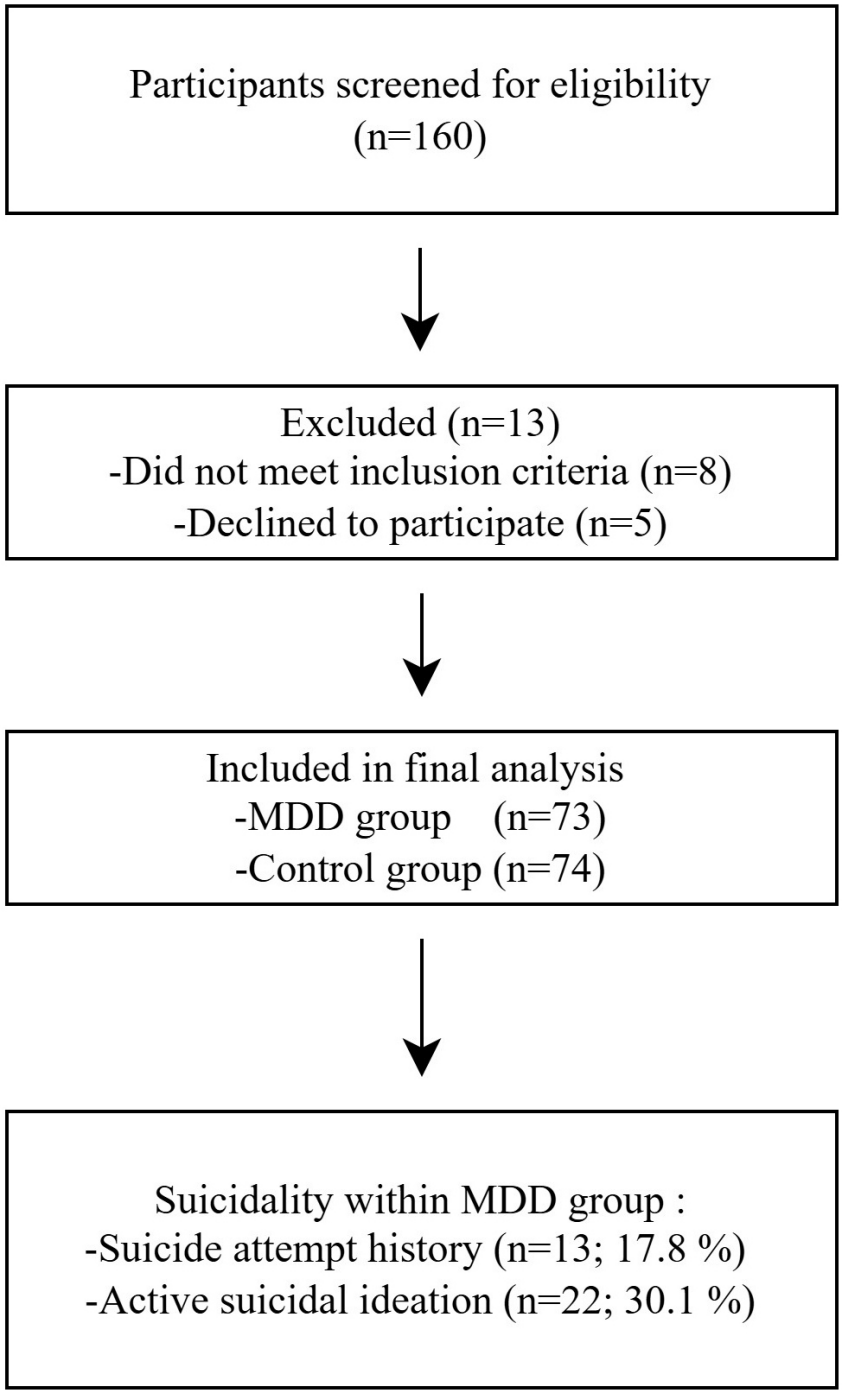
**Flow Diagram of Participant in the Case–Control Study**. MDD, Major depressive disorder.

A total of 160 individuals were screened for eligibility; 13 were excluded (eight not meeting inclusion criteria and five declining participation). Consequently, 147 participants were included in the final analysis, comprising 73 patients with MDD and 74 healthy controls. Among the 73 patients with MDD, 28 (38.4%) were first-episode, antidepressant-naive cases, and 45 (61.6%) were recurrent patients with a history of previous antidepressant treatment but medication-free for at least three months. Thirteen patients (17.8%) had a history of suicide attempt, and 22 patients (30.1%) exhibited current active suicidal ideation at the time of assessment (Fig. 1). The patient and control groups were comparable in age, sex, marital status, residence, and living status, while patients had lower educational attainment, higher unemployment, and greater smoking prevalence (Table 1, Ref. [35]).

**Table 1.** **Comparison of sociodemographic and clinical characteristics between patient and control groups**.

Variables		Patient (n = 73)	%	Control (n = 74)	%	*p*-value	Effect size
		n		n			
Sex						0.307	0.093
	Female	49	67.1	43	58.1		
	Male	24	32.9	31	41.9		
Marital Status						0.181	0.182
	Single	33	45.2	30	40.5		
	Married	33	45.2	42	56.8		
	Divorced/Widowed	7	9.6	2	2.7		
Education Level						<0.001*	0.287
	Primary	24	32.9	7	9.5		
	Secondary and Higher	49	67.1	67	90.5		
Living Status						0.393	0.070
	Living alone	19	26.0	24	32.4		
	Not living alone	54	74.0	50	67.6		
Residence						0.087	0.146
	Rural	17	23.3	9	12.2		
	Urban	56	76.7	65	87.8		
Employment Status						<0.001*	0.432
	Employed	32	43.8	63	85.1		
	Unemployed	41	56.2	11	14.9		
Occupation						-	-
	Civil Servant	12	16.4	48	64.8		
	Worker	12	16.4	11	14.9		
	Student	12	16.4	9	12.2		
	Tradesperson	5	6.9	2	2.7		
	Other	22	30.2	4	5.4		
	None	10	13.7	-	-		
BMI Group (n = 145)						0.652	0.067
	Underweight	5	6.8	3	4.2		
	Normal weight	33	45.3	37	51.4		
	Overweight	35	47.9	32	44.4		
Smoking Status						<0.001*	0.277
	Smoker	35	47.9	16	21.6		
	Non-smoker	38	52.1	58	78.4		

Note: Chi-square tests were used for categorical variables unless otherwise specified. Effect sizes (Cramér’s *V*) were interpreted as small (0.10), medium (0.30), and large (0.50) according to Cohen [35]. Occupational status was excluded from statistical analysis due to empty cells in certain categories. Significance is assumed for *p* < 0.05 and indicated by an asterisk (*). Body mass index (BMI) data were available for 145 participants; missing values for two individuals were due to incomplete anthropometric records.

Serum biomarker analyses revealed significantly lower Claudin-5 levels and elevated NLR, PLR, CRP, and INFLA scores in patients compared to controls, whereas MLR did not differ (Table 2, Ref. [35]). When patients were further divided into first-episode (antidepressant-naive) and recurrent subgroups, no significant differences were found in Claudin-5 or inflammatory indices, indicating that these alterations were consistent across both subgroups. Claudin-5 levels were not significantly associated with sociodemographic or clinical characteristics, although a nonsignificant trend toward higher values in mild depression was noted.

**Table 2.** **Comparison of serum Claudin-5 and inflammatory markers between patient and control groups**.

Variable	Group	Mean ± SD	Median	Min	Max	*p*-value	Effect size
Claudin-5 (ng/L)	Patients	1137.11 ± 748.73	797.30	401.30	3324.00	<0.001*	0.891
	Controls	1888.66 ± 936.64	1895.50	218.90	3363.00		
MLR	Patients	0.06 ± 0.08	0.02	0.00	0.40	0.144	0.106
	Controls	0.07 ± 0.11	0.02	0.00	0.50		
NLR	Patients	2.23 ± 1.11	2.00	0.90	8.90	<0.001*	0.829
	Controls	1.55 ± 0.53	1.50	0.80	3.60		
PLR	Patients	133.95 ± 47.39	123.40	64.00	277.00	0.032*	0.368
	Controls	118.25 ± 37.88	111.30	65.00	261.00		
CRP (mg/L)	Patients	3.10 ± 3.58	1.40	1.00	18.00	0.003*	0.571
	Controls	1.56 ± 1.81	0.70	0.00	9.00		
INFLA score	Patients	2.14 ± 5.42	2.00	–12.00	15.00	<0.001*	1.055
	Controls	–3.03 ± 4.38	–4.00	–7.00	4.00		

Note: Values are presented as (mean ± standard deviation) and median (range). Mann–Whitney *U* test was used for non-normally distributed variables. Effect sizes (Cohen’s *d*) were interpreted as small (0.20), medium (0.50), and large (0.80) according to Cohen [35]. MLR, monocyte-to-lymphocyte ratio; NLR, neutrophil-to-lymphocyte ratio; PLR, platelet-to-lymphocyte ratio; CRP, C-reactive protein; INFLA, inflammation Score. Significance is assumed for *p* < 0.05, and indicated by an asterisk (*).

PLR and INFLA scores correlated positively with HAM-D scores (r = 0.289, *p *= 0.013; r* =* 0.296, *p* = 0.011), but these associations did not remain significant after Bonferroni correction (adjusted α = 0.004). Serum Claudin-5 was not significantly associated with clinical or inflammatory variables (Table 3).

**Table 3.** **Correlations between clinical scores (HAM-D, BSSI) and biomarkers in the MDD group (n = 73)**.

Variables	HAM-D score	BSSI score
	Pearson *r* (*p*)	Pearson *r* (*p*)
Claudin-5 level	−0.084 (0.479)	−0.069 (0.560)
MLR	0.001 (0.996)	−0.003 (0.977)
NLR	0.207 (0.078)	0.079 (0.507)
PLR	0.289 (0.013)*	0.190 (0.107)
CRP	0.001 (0.992)	−0.085 (0.475)
INFLA score	0.296 (0.011)*	0.038 (0.752)

Note: Pearson correlation coefficient (*r*) and *p*-value (*p*) are given. Statistically significant correlations are indicated by an asterisk (*). HAM-D, Hamilton Depression Rating Scale; BSSI, Beck Scale for Suicide Ideation.

In additional analyses, none of the inflammatory indices showed significant correlations with BSSI Score, including MLR (r = –0.003, *p* = 0.977), NLR (r = 0.079, *p* = 0.507), PLR (r = 0.190, *p* = 0.107), CRP (r = –0.085, *p* = 0.475), and the INFLA score (r = 0.038, *p* = 0.752) (Table 3).

Patients with and without active suicidal ideation did not differ in Claudin-5 or inflammatory markers (Table 4, Ref. [35]).

**Table 4.** **Relationship between suicidal ideation and serum Claudin-5, inflammatory markers in the patient group (n = 73)**.

Variables	Active suicidal ideation present (n = 22)	Active suicidal ideation absent (n = 51)	*p*-value	Effect size (r)
	Median (min–max)	Median (min–max)		
Claudin-5 level	834.900 (401.300–3324.000)	774.900 (511.100–3183.000)	0.697	0.040
MLR	0.027 (0.000–0.310)	0.026 (0.000–0.460)	0.197	0.130
NLR	2.120 (1.200–8.900)	2.010 (0.900–4.200)	0.446	0.320
PLR	126.840 (94.000–277.000)	123.190 (64.000–260.000)	0.228	0.370
CRP	1.410 (1.000–10.000)	1.410 (1.000–18.000)	0.651	0.010
INFLA score	4.000 (−6.000–15.000)	2.000 (−12.000–12.000)	0.264	0.290

Note: Values are presented as median (min–max). Mann–Whitney *U* test was used to compare groups with and without suicidal ideation. Effect sizes (r) were interpreted according to Cohen [35], as small (0.10), medium (0.30), and large (0.50). *p* < 0.05 was considered statistically significant.

**Fig. 2.**
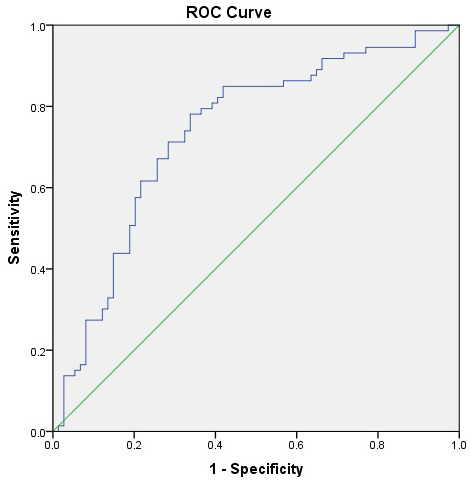
**ROC curve for Claudin-5 distinguishing patients with major depressive disorder (MDD) from healthy controls**. The AUC was 0.737 (95% CI: 0.655–0.819, *p* < 0.001), indicating fair discriminative ability. The optimal cut-off value was 1134.0 ng/L, with 71.2% sensitivity and 71.6% specificity.

**Fig. 3.**
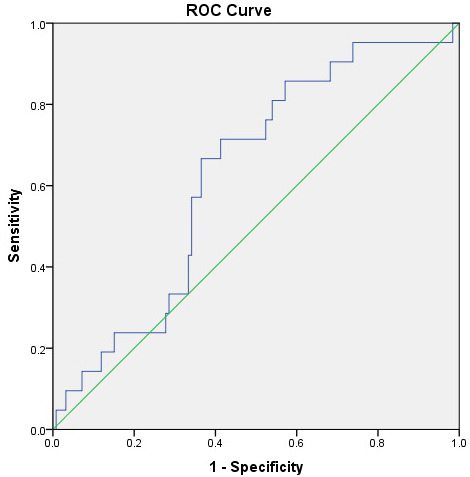
**ROC curve for Claudin-5 predicting the presence of suicidal ideation among patients with major depressive disorder (MDD)**. The AUC was 0.628 (95% CI: 0.512–0.744, *p* = 0.061), suggesting limited discriminative power for suicidal ideation.

ROC analysis demonstrated that Claudin-5 had fair diagnostic accuracy for distinguishing patients from controls (AUC = 0.737) (Table 5A, Ref. [36]) (Fig. 2), but limited value for predicting suicidal ideation (AUC = 0.628) (Table 5A, Fig. 3). When Claudin-5, NLR, and INFLA scores were entered together into a composite ROC model, the model retained fair diagnostic accuracy (AUC = 0.720, *p* < 0.01), suggesting that inflammatory and neurovascular markers may provide complementary—rather than superior—predictive information (Table 5B). Logistic regression indicated that higher NLR and INFLA scores, but not Claudin-5 or family history, were independent predictors of suicidal ideation (Table 6). VIF values for the NLR and INFLA scores were all below 2.0 (range: 1.12–1.68), indicating no evidence of multicollinearity among the predictors.

**Table 5A.** **Discriminative ability of serum Claudin-5 levels for major depression and suicidal ideation: ROC curve analysis**.

Risk factor	Outcome	AUC (95% CI)	Cut-off	*p*-value	Sensitivity/Specificity
Claudin-5	Depression Presence	0.737 (0.655–0.819)	1134.0	<0.001*	71.2%/71.6%
Claudin-5	Suicidal Ideation	0.628 (0.512–0.744)	910.25	0.061	61.9%/63.5%

Note. Claudin-5 levels were tested for discriminating major depressive disorder vs. control status and the presence of suicidal ideation. Optimal cut-off values were determined using Youden’s index. Significance is accepted for *p* < 0.05 and indicated with an asterisk (*). AUC values were interpreted as 0.5–0.6 = fail, 0.6–0.7 = poor, 0.7–0.8 = fair, 0.8–0.9 = good, and >0.9 = excellent according to Hanley and McNeil [36]. AUC, area under the curve; CI, confidence intervals.

**Table 5B.** **Diagnostic accuracy of composite biomarker model (Claudin-5 + NLR + INFLA score) for distinguishing MDD from controls**.

Model	AUC (95% CI)	*p*-value	Sensitivity/Specificity
Claudin-5 + NLR + INFLA	0.720 (0.637–0.803)	<0.01	72.5%/70.3%

Note. Composite model derived from binary logistic regression using predicted probabilities.

**Table 6.** **Multivariate logistic regression analysis predicting suicidal ideation**.

Variable	β (Standardized)	95% CI	*p*-value
Claudin-5 (ng/L)	–0.145	−0.312–0.025	0.091
NLR	0.232	0.045–0.382	0.011*
INFLA Score	0.198	0.014–0.332	0.037*
Family History (Yes/No)	0.102	−0.034–0.229	0.131

Note. Standardized beta coefficients (β), 95% CI, and *p*-values are shown. Significance is assumed at *p* < 0.05 and is indicated with an asterisk (*). Variance Inflation Factors (VIF) for neutrophil-to-lymphocyte ratio (NLR) and inflammation (INFLA) score ranged from 1.12 to 1.68, indicating no multicollinearity.

## 4. Discussion

This study demonstrated that serum Claudin-5 levels were significantly lower in patients with MDD who had either never used antidepressants or had been medication-free for at least three months, while no significant associations were found between Claudin-5 and symptom severity, suicidal ideation, or inflammatory markers. Beyond statistical significance, several group differences—particularly the reductions in Claudin-5 and elevations in NLR—showed medium-to-large effect sizes, suggesting that these alterations may hold clinical as well as statistical significance. To the author’s knowledge, this is the first study to assess serum Claudin-5 alongside inflammatory indices and suicidal ideation in untreated adults with MDD, providing new insights into its neurovascular pathophysiology.

Groups were matched for age, sex, and body mass index (BMI), reducing demographic confounding. Although unemployment and smoking were slightly higher in MDD, these factors are unlikely to explain the biochemical differences observed.

Claudin-5, which is crucial for BBB integrity, has been implicated in several neuropsychiatric disorders, including MDD [12, 13, 16]. These findings align with recent evidence on tight-junction proteins in psychiatry: A 2025 systematic review reported altered Claudin-5 and occludin levels across major psychiatric disorders, supporting BBB involvement beyond single-study findings and diagnostic categories [24].

The findings reported here of reduced serum Claudin-5 levels are consistent with previous postmortem and preclinical studies showing downregulation in the hippocampus and nucleus accumbens of depressed individuals [12, 13]. A drug-free cohort minimized medication effects; elevated Claudin-5 in medicated or adolescent samples, and likely reflects treatment or developmental influences [37, 38, 39]. Additionally, genetic and molecular vulnerabilities involving Claudin-5 and other blood–brain barrier–related pathways have been associated with cognitive and affective disturbances, suggesting a broader etiological role for tight-junction dysregulation in mood disorders [16, 39, 40].

No significant associations were found between serum Claudin-5 levels and depression severity, suicidal ideation, or duration of untreated symptoms. This suggests that Claudin-5 reflects relatively stable cerebrovascular alterations rather than acute, state-dependent changes. While imaging and experimental studies have reported BBB permeability changes correlating with symptom severity, peripheral markers such as Claudin-5 may only partially reflect such central neurovascular dynamics [11, 40, 41]. The BBB is a complex multicellular structure involving pericytes, astrocytes, and immune modulators; thus, single protein measures may not capture its full functional integrity.

The absence of correlation between Claudin-5 or inflammatory indices and depression severity may reflect their different temporal and biological characteristics. Claudin-5 may represent a relatively stable marker of endothelial integrity, indicating a persistent neurovascular vulnerability, whereas systemic indices such as NLR, PLR, and MLR are state-dependent and fluctuate with short-term physiological or psychosocial stress. Their lack of association with depression severity in this cross-sectional sample may reflect temporal immune variability and depressive heterogeneity rather than a lack of pathophysiological relevance. Overall, these findings suggest that Claudin-5 and inflammatory markers capture distinct yet complementary dimensions of neurovascular and immune dysregulation in MDD [3, 40, 42].

The current findings are in line with several previous studies reporting reduced Claudin-5 expression in both clinical and preclinical models of depression. For instance, Menard *et al*. [13] and Dion-Albert *et al*. [12] demonstrated stress-induced downregulation of Claudin-5 in animal models and in unmedicated depressed individuals. Similarly, lower circulating levels of other tight-junction proteins such as occludin and zonulin have been observed in mood and psychotic disorders [17, 24]. Conversely, studies involving medicated or adolescent samples have sometimes found elevated Claudin-5, likely reflecting treatment or developmental effects [24, 37, 38]. Together, these findings reinforce the hypothesis that endothelial dysfunction and altered BBB integrity are common biological features across major psychiatric conditions.

Mechanistically, chronic inflammation may contribute to BBB disruption through cytokine-mediated pathways. Experimental studies have shown that pro-inflammatory cytokines such as IL-6 and TNF-α down-regulate tight-junction proteins, including Claudin-5 and occludin, leading to endothelial barrier dysfunction and increased permeability [43, 44]. Such cytokine-induced alterations enhance peripheral-to-central immune signaling and promote microglial activation**,** thereby linking systemic inflammation to neuroinflammatory processes observed in major depressive disorder. These mechanisms are consistent with the neurovascular-inflammatory model of depression, which proposes that sustained low-grade inflammation compromises BBB integrity and neuronal homeostasis [3, 45].

Although PLR and INFLA scores showed positive correlations with depression severity, these associations did not remain significant after Bonferroni correction, suggesting that they should be interpreted as trend-level findings. Future studies with larger samples are needed to confirm these relationships.

To further contextualize Claudin-5 within neuroimmune mechanisms and its relationship with inflammatory markers, it was found that MDD patients exhibited significantly elevated NLR, PLR, CRP, and INFLA scores compared to healthy controls (*p* < 0.001, 0.032, 0.003, and <0.001, respectively), supporting the low-grade inflammation model of depression [2, 3, 42]. However, Claudin-5 level was not significantly correlated with any of these markers. This finding is in line with the downregulation hypothesis, which proposes that reduced Claudin-5 expression may arise from transcriptional repression or endothelial dysfunction within the CNS, rather than from passive leakage caused by systemic inflammation. No significant correlations were found between serum Claudin-5 and inflammatory indices, possibly reflecting a complex, non-linear interaction between systemic inflammation and BBB integrity. Notably, the INFLA-score, as a composite index integrating both humoral (CRP) and cellular inflammatory components, has been shown in large epidemiologic cohorts to correlate with depressive symptomatology and explain part of the association between chronic low-grade inflammation and depression severity [33, 34]. In this context, findings reported here of higher INFLA-scores in MDD support its potential as an integrative biomarker reflecting the cumulative inflammatory burden relevant to depressive pathophysiology. Moreover, cytokine-mediated pathways, particularly involving TNF-α and IL-6, disrupt Claudin-5 expression in a region- and time-dependent manner [24, 46]. Compensatory regulation of other tight-junction proteins or individual variability in immune responses may also mask peripheral–central associations, suggesting that Claudin-5 changes in MDD may reflect a parallel rather than a directly proportional pathway to inflammation.

Hematologic indices such as the NLR and PLR are non-specific and may be influenced by lifestyle or metabolic factors (e.g., smoking, obesity, stress). Although smoking and unemployment were more frequent in the MDD group, these factors did not significantly affect inflammatory marker or Claudin-5 levels, and the group difference in Claudin-5 remained significant after accounting for them. Still, residual confounding cannot be excluded, and future studies should control for such variables.

Experimental studies indicate that inflammatory cytokines such as TNF-α and IL-6 may suppress Claudin-5 expression via the NF-κB and HDAC1 signaling pathways [47, 48], yet the current results did not support a direct association between systemic inflammation and Claudin-5 levels. Two mechanistic models have been proposed: The spillover model, which posits that BBB disruption leads to Claudin-5 leakage into peripheral circulation [11, 25], and the downregulation hypothesis, which attributes reduced serum levels to impaired expression at the endothelial level [12, 13]. The current findings—specifically, the lower Claudin-5 levels in MDD and lack of correlation with inflammatory indices—lend stronger support to the latter.

Overall, Claudin-5 appears to be a promising but complex biomarker of neurovascular dysfunction in MDD. Its peripheral concentrations may reflect more stable endothelial traits rather than fluctuating clinical states, and its role should be explored further through multimodal and longitudinal research integrating central and peripheral assessments.

Subgroup analysis comparing first-episode and recurrent MDD patients showed no significant differences in Claudin-5 or inflammatory indices (MLR, NLR, PLR, CRP, INFLA score). These results suggest that neurovascular and inflammatory alterations in MDD are not strongly affected by illness chronicity or recent treatment, supporting Claudin-5 as a relatively stable endothelial marker in untreated MDD.

Although this is the first study to assess Claudin-5 in drug-free adult MDD patients alongside inflammatory markers and suicidal ideation, the findings reported here suggest that Claudin-5 alone has limited predictive value as a biomarker of suicide risk. In ROC analysis, serum Claudin-5 demonstrated moderate accuracy in differentiating MDD patients from healthy controls (AUC = 0.737, *p* = 0.001), aligning with earlier findings in adolescent populations [38]. However, its ability to predict suicidal ideation was weak (AUC = 0.628), suggesting that Claudin-5 alone may not significantly serve as a reliable standalone marker for suicide risk assessment.

Multivariate logistic regression revealed that NLR and INFLA score were significant independent predictors of suicidal ideation, consistent with prior research highlighting systemic inflammation as a key contributor to suicide vulnerability [18, 25]. Consistent with these multivariable results, a recent meta-analysis has shown that immune-related biomarkers differ between individuals with and without suicidal behaviors, reinforcing the clinical relevance of systemic inflammation in suicide vulnerability [25, 46]. Interestingly, although NLR and INFLA score did not show significant differences between patients with and without suicidal ideation in univariate comparisons, both emerged as independent predictors in multivariate regression. This discrepancy likely reflects the fact that regression analysis accounts for potential confounders and reveals underlying associations that simple group comparisons may obscure. In this context, systemic inflammation may exert a subtle but clinically meaningful influence on suicidal vulnerability, which becomes more evident when considered alongside other biological and clinical variables. These findings emphasize the importance of using multivariable approaches to capture the complex interplay between inflammation and suicidality in MDD.

Importantly, when Claudin-5 was combined with NLR and INFLA score in a composite biomarker model (Table 5B), the model retained fair diagnostic accuracy (AUC = 0.720, *p* < 0.01), suggesting that neurovascular and inflammatory markers may provide complementary rather than superior predictive information. Although serum Claudin-5 alone showed weak predictive power, its inclusion in composite biomarker panels may still contribute distinct biological value in clinical stratification.

Given their low cost and accessibility, NLR and INFLA score could be readily integrated into clinical risk assessment protocols for suicide in psychiatric settings. Moreover, the inclusion of Claudin-5 alongside inflammatory markers may enhance the biological specificity of such models. Emerging research on gut–brain–microbiome interactions suggests that peripheral barrier dysfunction (“leaky gut”) may interact with BBB disruption and inflammatory pathways, further linking systemic and neurovascular processes in depression and suicidality [8, 40, 49]. Future prospective and neuroimaging studies are needed to further explore the combined role of Claudin-5 and inflammatory indices in MDD. If validated in future studies, Claudin-5 and inflammatory indices such as NLR and INFLA score could potentially serve as peripheral biomarkers for refining biological subtypes of depression and identifying patients at heightened suicide risk.

This study has several strengths, including drug-naive or medication-free MDD patients, which allowed an unconfounded assessment of peripheral Claudin-5 levels. The use of age- and sex-matched controls improved internal validity, while simultaneous evaluation of depressive severity, suicidality, and inflammatory indices provided a multidimensional view of Claudin-5’s clinical relevance. Together, these findings support its role in neurovascular dysregulation and its potential as a peripheral marker of BBB dysfunction in depression.

However, several limitations warrant consideration. The cross-sectional design precludes causal inference, and serum Claudin-5 may not fully represent central levels. Neuroimaging methods such as dynamic contrast-enhanced MRI were not used to confirm BBB permeability. Although the total sample size was adequate for medium effects, subgroup analyses (e.g., suicidality) were likely underpowered; detecting small-to-moderate effects (d = 0.3, 80% power, α = 0.05) would require ~175 participants per group. Thus, null subgroup findings should be interpreted cautiously. Additionally, some patients had prior depressive episodes, potentially introducing residual confounding, and lifestyle or environmental factors (e.g., diet, microbiota) were not controlled.

## 5. Conclusion

This study provides preliminary but important evidence that serum Claudin-5 levels are significantly reduced in untreated patients with MDD, possibly indicating relatively stable endothelial alterations rather than acute, state-dependent changes. While Claudin-5 alone showed limited utility for predicting symptom severity or suicide risk, peripheral inflammatory markers such as NLR and INFLA scores were independently associated with suicidal ideation. Although the composite biomarker model combining Claudin-5 with inflammatory indices did not improve predictive performance compared with Claudin-5 alone, these markers may offer complementary biological information reflecting distinct neurovascular and inflammatory mechanisms. Future longitudinal and multimodal studies are warranted to confirm these findings and to develop integrated biomarker approaches for depression and suicidality.

## Disclosure

This article is based on the medical specialty thesis titled “Comparison of Serum Claudin-5 Levels in Patients with Major Depressive Disorder and Healthy Controls: Evaluation of the Relationship Between Serum Claudin-5 Levels, Suicidal Ideation, Anxiety Severity, and Systemic Inflammatory Parameters”, conducted by Nurbanu Keskin at Muğla Sıtkı Koçman University Training and Research Hospital under the supervision of primary advisor Mahmut Selçuk and secondary advisor Ercan Saruhan.

## Availability of Data and Materials

The data supporting the findings of this study are available from the corresponding author upon reasonable request.
